# Language extinction and linguistic fronts

**DOI:** 10.1098/rsif.2014.0028

**Published:** 2014-05-06

**Authors:** Neus Isern, Joaquim Fort

**Affiliations:** 1 Quantitative Archaeology Laboratory, Departament de Prehistòria, Universitat Autònoma de Barcelona, 08193 Cerdanyola del Vallès, Spain; 2 Complex Systems Laboratory, Departament de Física, Universitat de Girona, 17071 Girona, Catalonia, Spain

**Keywords:** language competition, reaction–diffusion, fronts, language extinction, cultural transmission

## Abstract

Language diversity has become greatly endangered in the past centuries owing to processes of language shift from indigenous languages to other languages that are seen as socially and economically more advantageous, resulting in the death or doom of minority languages. In this paper, we define a new language competition model that can describe the historical decline of minority languages in competition with more advantageous languages. We then implement this non-spatial model as an interaction term in a reaction–diffusion system to model the evolution of the two competing languages. We use the results to estimate the speed at which the more advantageous language spreads geographically, resulting in the shrinkage of the area of dominance of the minority language. We compare the results from our model with the observed retreat in the area of influence of the Welsh language in the UK, obtaining a good agreement between the model and the observed data.

## 1. Introduction

Mathematical and computational models are currently applied to many cross-disciplinary studies in areas such as ecology [1–3], archaeology [4–6] or linguistics. In linguistics, studies have been undertaken to model the internal evolution of languages [7,8] as well as the geographical processes of language competition and replacement [9,10]. In this paper, we focus on the latter problem.

Language evolution takes place at a rather slow rate, with a timescale of about a thousand years for a single language to evolve into several different languages [11]. However, language death is a process that takes place at substantially faster rates [12]. Language death usually involves language shift to a new dominant language [12] (either imposed [11] or acquired from neighbouring contact [13]), and the language eventually dies with its last speaker [14].

Language birth and death are natural ongoing processes worldwide, but, in recent times, the processes of language extinction have accelerated, partly owing to improved communications and globalization processes [15,16]. Currently, about 4% of the languages are spoken by 96% of the population, whereas 25% of the languages have fewer than 1000 speakers [14]. In addition, unless current trends change, linguists estimate that 90% of the about 6000 languages currently spoken may become extinct, or greatly endangered, by the end of this century [17].

The main driver for the current processes of language shift is the perception of a potential economic improvement [15,16,18]. This results in speakers of minority languages ceasing to speak their language and, most importantly, to transmit it to their children, in favour of neighbouring (usually co-official) languages regarded as socially and economically more advantageous [15–17].

In 2003, Abrams & Strogatz [19] proposed a simple two-language competition model, with preferential attachment to one of the languages, to mathematically describe the dynamics of language shift. Their model describes the rate of change in the population fraction of two linguistic groups, *A* and *B*, as follows1.1
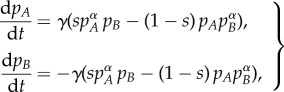
where *p_A_* and *p_B_* are the fractions of the total population corresponding to each linguistic group, with *p_B_* = 1 − *p_A_*, *s ∈* (0, 1) is a quantification of the status of language *A* (and consequently 1 − *s* is the status of language *B*), *γ* is a parameter that scales the time and *α* determines the relative importance of language *A* over *B* in attracting speakers [19].

Abrams & Strogatz [19] applied equation (1.1) to explain historical data on language decline for the Quechua, Welsh and Scottish languages. In such applications, they chose *p_A_* as the fraction speaking only the high-status language (Spanish for the first case, and English for the later ones), and *p_B_* as the fraction of the population that can speak the low-status language (either as monolinguals or bilinguals^1^), and observed that the best fit to the historical data, if no external changes were introduced, yielded the extinction of the low-status language. Alternatively, Abrams *et al.* [20] showed that equation (1.1) can also be applied to model the evolution of other competing cultural traits, particularly the observed decline in religious affiliation.

Other authors have extended or taken a different approach to the study of equation (1.1) when applied to language competition. Mira & Paredes [21] extended the same non-spatial model by including a similarity parameter to study the evolution of three population groups, two monolingual and one bilingual, when two similar languages are competing. In their model, the decrease in monolinguals of the low-status language is due to an increase of both bilinguals and monolinguals of the high-status one. Alternatively, Stauffer *et al.* [22] applied a simplified version of equation (1.1), with *α* = 1, using an agent-based model, and obtained qualitatively good agreement with the analytic model except in the case of socially equivalent languages.

On the other hand, some authors have extended equation (1.1) by including spatial dynamics in the study of language competition. Patriarca & Leppännen [23] included equation (1.1) as part of a reaction–diffusion model and studied the evolution of two languages initially located at two adjacent regions, obtaining a stable area of coexistence near the border. This result was possible by assuming a barrier that restricted linguistic influence of an individual only to the individuals located at the same region. Later, Patriarca & Heinsalu [24] extended the analysis of this spatial model by studying the effect of different initial distributions of the two languages on which language becomes extinct (without barriers). They also considered the effect of a barrier that diminishes dispersal between separated regions (instead of affecting the interaction as in reference [23]) and found that this kind of barrier also makes it possible for each language to survive on each side of the barrier (when the barrier is restrictive enough). By contrast, Fort & Pérez-Losada [25] used equation (1.1) in an integro-difference equation with non-coupled population growth, and applied it to predict the speed of the Welsh language replacement, finding reasonably good agreement.

**Figure 1.**
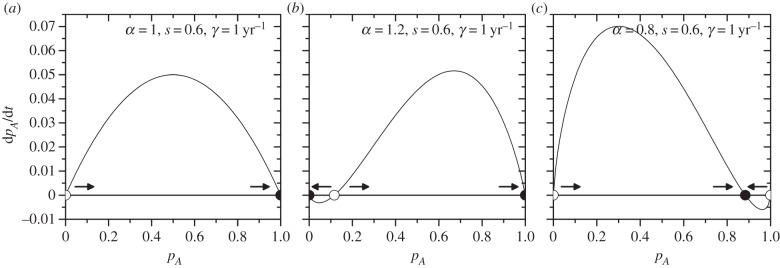
Variation of the fraction of speakers of the high-status language for the linguistic model by Abrams & Strogatz (equation (1.1) and reference [19]) for different values of the parameter *α*. (*a*) Shows the behaviour when *α* = 1, (*b*) when *α* > 1 (*α* = 1.2), and (*c*) when *α* < 1 (*α* = 0.8). Circles correspond to the fixed points, with stable points represented by filled circles, and the unstable ones by empty circles. The arrows show the natural evolution of *p_A_* towards or away from the fixed points. In all cases, *s* = 0.6 and *γ* = 1 yr^−1^.

However, the studies mentioned above use some standard or mean values for the parameters in equation (1.1), but when one examines explicitly the results for the datasets in reference [19], one finds that equation (1.1) presents some limitations when extrapolating these results. As an example, the best fit for the Quechua population (figure 1*b* in reference [19]) is *γ* = 0.147 yr^−1^, *α* = 1.98, *s* = 0.74, and these parameter values imply that if in a region the fraction of Quechua speakers is higher than 75% (*p_A_* < 0.25), it would be the Spanish speakers who would learn Quechua (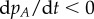). Obviously, such dynamics would have avoided the observed replacement of Quechua by Spanish [16,26]. Similarly, the best fit for the Welsh language in all of Wales (fig. 1d in reference [19]) is *γ* = 0.144 yr^−1^, *α* = 0.92, *s* = 0.57. Then, according to equation (1.1), language shift would be reversed (English speakers would start speaking Welsh, i.e. 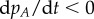), once the fraction of English speakers reaches about 97% of the population (*p_A_* > 0.97). Again, such behaviour disagrees with historical tendencies [17].

In order to solve this problem, we note that linguistic studies indicate that language shift happens mainly towards high-status languages, whereas the speakers of high-status languages (almost) never learn the minority language [12,27] (the few who do are often ‘intellectuals from the city’ [16]). For this reason, in this paper, we develop a simpler model of language shift, allowing only for speakers of the low-status language to shift to the high-status one (but not high-status speakers to shift to the low-status language).

One of the consequences of language replacement through neighbouring language acquisition is the progressive retreat of the language frontier, with the consequent shrinkage of the area where the minority language is spoken [26,28,29]. In this paper, we apply our new language shift model to describe such situations and explain the rate at which the higher-status language expands geographically and replaces the indigenous language. To do so, we introduce a reaction–diffusion system to describe the spatial evolution of both competing languages, including an interaction term to describe the language shift dynamics. The analysis of this system allows us to infer the speed at which a more advantageous language overcomes the dominance of a minority language, leading it to a process of possible extinction. We apply our new non-spatial model to the same datasets as in reference [19] to establish its validity, and then implement it to estimate the speed of linguistic fronts. We study the sensitivity of the model to the linguistic parameters using realistic values, and then compare the model predictions with the observed front of retreat of the Welsh language. Finally, we discuss the conclusions and implications of our results.

## 2. Methods

### 2.1. Limitations of the Abrams–Strogatz model

Before introducing our new approach, we further analyse the role of the parameters and fixed points in the model by Abrams & Strogatz [19], equation (1.1), and see the reason for the extrapolation problems mentioned in §1 from a formal point of view.

The parameter *γ* in equation (1.1) is a scale factor and, as such, it does not play a role in determining the final outcome in the linguistic competition, but it modifies only the rate at which the evolution takes place.

The final outcome of the linguistic competition defined by equation (1.1) is determined by the fixed points (or equilibrium points), which depend then on the values of *s* and *α*. The fixed points p*_A_ are those that fulfil the equation d*p_A_*/d*t* = 0. The values p*_A_ = 0 and 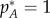 (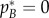) are trivial solutions of this equation, and thus fixed points. In addition, when *α ≠* 1, there is a third fixed point given by2.1
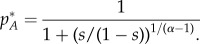


This expression can be obtained using the fact that 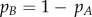 in the condition d*p_A_*/d*t* = 0.

If we now look at the stability, then a fixed point is stable when, if the system is perturbed away from this fixed point, then it returns to it (see arrows in figure 1). Therefore,2.2
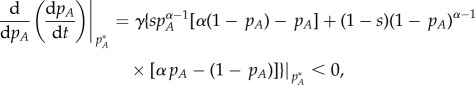
where, again, we have used that *p_B_* = 1 − *p_A_*. We have different stability scenarios depending on the value of *α*.

- If *α* = 1, then the system has only two fixed points 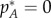 and 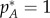 (in this case, equation (2.1) has no solution) and the stability condition, equation (2.2), is reduced to2.3
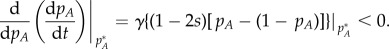
Then, we see that when *s* > 0.5—that is, when *A* has a higher status—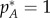 is stable (it fulfils the condition in equation (2.3)) and 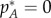 is unstable. Therefore, for any initial distribution of population fractions, group *A* eventually gains all the speakers and *B* becomes extinct (this scenario is shown in figure 1*a*). The opposite happens when *s* < 0.5, with p*_A_ = 0 being the stable point. If *s* = 0.5, then both linguistic groups are socially equivalent, and the population fraction does not change over time.
- If *α* > 1, then we can see that equation (2.2) holds for both 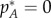 and 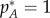, for any value of *s*. Therefore, they are both stable fixed points and the third fixed point, given by equation (2.1), is necessarily unstable (figure 1*b*). Then, the extinction or prevalence of group *A* depends on whether the initial population fraction *p_A_* is lower or higher than the unstable fixed point respectively.
- If *α* < 1, 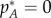 and 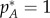 are both unstable points for any value of *s*. This can be observed more easily if the stability condition is written as2.4
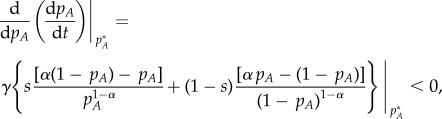
where we can see that for both extreme values the sum within the curly brackets in equation (2.4) is +*∞*. Then, the third fixed point given by equation (2.1) is necessarily stable (figure 1*c*), and therefore this is the final population fraction for group *A*, for any initial distribution with presence of individuals of both groups.

So, if we now go back to the real population data from reference [19] mentioned in §1, then we see that for the Quechua population (*α* = 1.98, *s* = 0.74), there is an unstable fixed point at 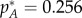 (given by equation (2.1)). Therefore, according to the model, only if the initial population fraction of Spanish speakers is higher than this value will their relative fraction grow, which we have seen to be historically unreasonable. On the contrary, for the case of the Welsh language (*α* = 0.92, *s* = 0.57), p*_A_ = 0.971 is a stable fixed point. This would signify a long-term coexistence of both linguistic groups once the fraction of Welsh speakers is around 3%. Even though we cannot deem it impossible, such behaviour seems historically unreasonable without segregation or application of linguistic policies, which are not included in the model.

### 2.2. Basic model

In this paper, we want to model the language shift between two competing languages, *A* and *B*, with language *A* being regarded as socially and economically more advantageous and attractive than the other language (*B*). We define the population fraction *p_A_* as monolingual speakers of the high-status language *A*, and *p_B_* corresponds to the fraction of the population able to speak language *B. p_B_* can include both monolingual or bilingual speakers of language *B* (as in the data used in reference [19]).

We use a two-language population model, such as in references [19,22–25], rather than explicitly including bilingual populations [21,30,31], because we consider that dividing the population between speakers and non-speakers of the low-status language can provide a good enough scenario of the health and evolution of endangered languages. Besides, often, there is no official information on multilingual speakers to compare with a more sophisticated model (such as in the Peruvian census [32]).

We want to define a model that can be applied to current-day situations where minority regional languages are in competition with (often co-official) languages that have a higher status, and usually a wider area of influence. Therefore, in accordance with historical data [17], we assume a simplified situation where the language shift can happen only towards the high-status language. This would correspond to a scenario where the relative status does not change significantly over time (e.g. no efficient language policies are applied).

We propose, then, the following model with which we have found the best agreement with historical data2.5
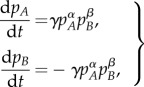
where *γ* is a time-scaling parameter, and *α*, *β* ≥ 1 are two parameters related to the attraction or perceived value of both languages: we may regard *α* ≥ 1 as a measure of the difficulty of language *A* to attract speakers (recall that *p_A_* ≤ 1), and *β* ≥ 1 as the resistance of language *B* to lose speakers. In this new model, the right-hand side is the same for both languages, but they have opposed signs. Therefore, language *A* gains the same number of speakers that language *B* loses per unit time.

If, similar to §2.1, we analyse the fixed points in this new model, we can see that only 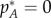 and 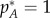 (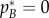) can be fixed points, i.e. can fulfil the condition d*p_A_*/d*t* = 0. Because equation (2.5) only allows for *p_A_* to increase over time and *p_B_* to decrease, the only stable point in this case is p*_A_ = 1. Therefore, in this model, the community speaking language *B* will eventually disappear in benefit of the monolingual speakers of language *A*.

We have fitted our model to the experimental data in reference [19] by integrating equation (2.5) using a fourth-order Runge–Kutta method [33]. We have determined the best set of parameters using a least-squared error approach (see §3.1).

### 2.3. Reaction–diffusion model and numerical integration

In order to model the geographical dynamics of both languages and estimate the expanding speed of the language replacement front, we have applied equation (2.5) as an interaction term in a reaction–diffusion system. To reformulate this term for population densities, we have applied that 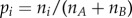 for *i* = *A*, *B* (where *n_A_* and *n_B_* are, respectively, the population densities of speakers of languages *A* and *B*) and assumed that the variation of the total population density over time can be neglected when compared with the variation of each subgroup (this is a realistic assumption at least for the Welsh, Scottish Gaelic and Quechua populations considered in this paper [16,35]). This yields2.6
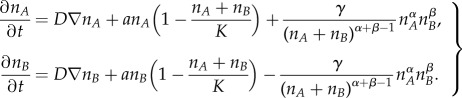


The first term on the right-hand side is the diffusive term, with *D* the diffusion coefficient. The second term is a logistic growth term for two competing populations, where the limiting term takes into account the presence of the other population, because they share the same space and resources and have the same carrying capacity *K* [24,34]. The last term is the language shift term from equation (2.5) for population densities rather than population fractions, as required in order to model diffusion [34] (left-hand side and first term on the right-hand side).

Because the frontier between competing languages, at least for the cases considered here, is mostly planar shaped (see maps in references [16,17,28,30]), we can assume planar fronts. Then, we can choose the *x*-axis parallel to the front speed, and therefore *p_A_*(*x*, *y*, *t*) does not depend on *y*. This simplifies equation (2.6) into the following one-dimensional system2.7
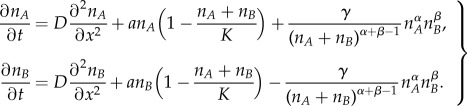
In order to study the system evolution, we have discretized and integrated numerically the system (2.7) on the nodes of a grid, by using an implicit method [33]. This requires specifying the initial conditions. Thus, we have assumed that initially the two-dimensional region corresponding to an interval of *x* (e.g. one-fifth of the complete range of *x*, located at the left-hand side) is occupied only by speakers of language *A* (*n_A_* = *K* and *n_B_* = 0), and the rest of the space is occupied by speakers of language *B* (*n_A_* = 0 and *n_B_* = *K*). The numerical integration of the set of equations (2.7) displays a front of *A*-speakers that expand their range into the region of *B*-speakers, and travels together with a retreating front of *B*-speakers. This allows us to find the front position at each timestep (defined, for example, as the value of *x* such that 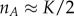). The slope of the front position versus time yields the front speed.

For modern examples of language substitution, populations display fairly constant densities in time (near the carrying capacity). Then, if we assume that the total population density is at approximately the carrying capacity, 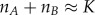, the population growth term (second term in equations (2.6) and (2.7)) becomes negligible (and in fact, our numerical integrations yield the same results with or without this term). Therefore, we obtain a much simpler system that, after dividing both equations by the carrying capacity *K*, can be written as follows2.8
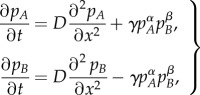
where the system is now expressed in terms of the population fractions (thus now *p_i_* = *n_i_*/*K* for *i* = *A*, *B*).

### 2.4. Variational analysis and bounds for the front speed

Besides the results from the numerical integration, it is possible to derive analytical bounds for the front speed. To do so, we first generalize equation (2.8) by using dimensionless variables 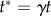 and 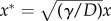. In addition, because we are now using population fractions we can apply that *p_B_* = 1 − *p_A_* and we can describe the system with a single equation2.9
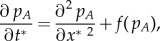
where 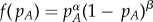. As 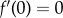, we cannot apply linear or marginal stability analysis [36], but it is still possible to find some constrains to the front speed by resorting to variational analysis. Using the variational approach described by Benguria and Depassier, it has been shown that, if *f*(*p_A_*) > 0 for 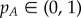, the following expression provides a lower bound for the front speed (see reference [37], equation (10))2.10
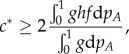
where *g*(*p_A_*) is and arbitrary positive function and 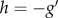. Because equation (2.10) must hold true for any function *g*, the one that yields a larger lower bound is the best one [38]. As usual, we consider the set of lower bounds given by the series of trial functions 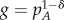, with 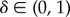 [38,39]. Solving the integrals in equation (2.10) for these trial functions and 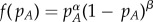, we find the following lower bound for the front speed of the language expansion2.11
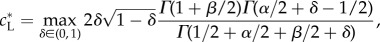
where the gamma function is defined by the following integral 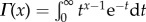, for *x* > 0 [40].

It has been shown that it is possible to find a function 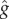 (which is not analytically manageable) for which the equality in equation (2.10) holds, thus the front speed is [38,39]2.12
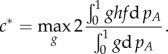
We can now use equation (2.12) to obtain an upper bound for the front speed by applying Jensen's inequality and integrating by parts [38,39], which yields the following expression (see reference [38], last equation for *ϕ* = 0; or reference [39], equations (27)–(28) for *a* = 0)2.13
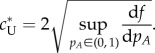


And when applying equation (2.13) to our interaction term, 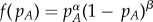, we obtain that the upper bound for the linguistic front speed is given by2.14
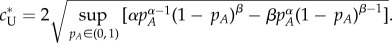


With these two analytic expressions, equation (2.11) and (2.14), we obtain a range that contains the exact speed for the linguistic front (the exact speed can be estimated by the numerical integration, as described in §2.3). For given values of *α* and *β*, the explicit result for the lower bound is obtained by searching for the maximum value of the right-hand side in equation (2.11) for values of *δ* within the interval (0, 1), and the upper bound is found analogously with equation (2.14) and values of *p_A_* within the interval (0, 1).

Note that this same analysis would not yield a lower or an upper bound for the Abrams–Strogatz model (equation (1.1)), because this technique requires that *f*(*p_A_*) > 0 for *p_A_∈*(0, 1) and as seen in §2.1 and figure 1, this condition is not always fulfilled for equation (1.1).

## 3. Results

### 3.1. Non-spatial model

**Figure 2.**
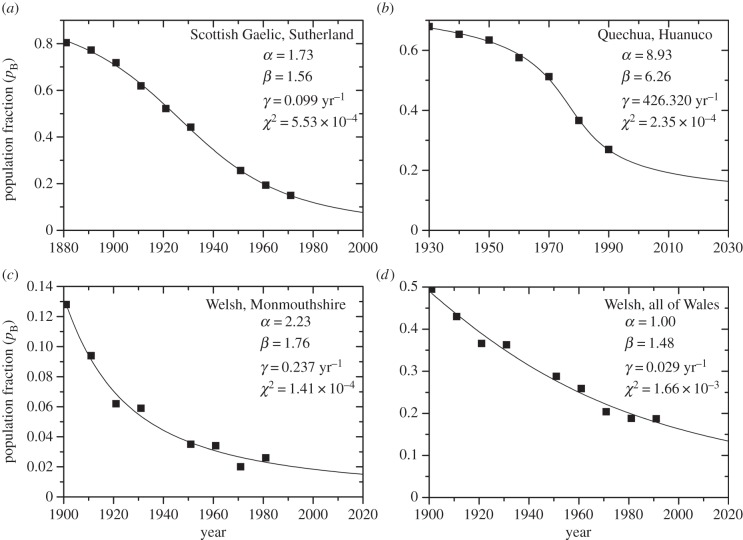
(*a*–*d*) Decline over time of the population fraction of speakers of minority languages. Symbols correspond to historical data obtained from reference [19]. Lines correspond to the best fit to the historical data obtained with equation (2.5). The corresponding languages, best-fit values of the parameters *α*, *β* and *γ*, and the value of *χ*^2^ from the least-squares regression are shown on the plots.

In this paper, we have proposed a new interaction term to model the dynamics of language competition, equation (2.5). Figure 2 shows the results of fitting this model to the decline of three languages in the four following regions (data from the plots in reference [19]): (i) Scottish Gaelic in Sutherland, Scotland, (ii) Quechua in Huanuco, Peru, (iii) Welsh in Monmouthshire, and (iv) Welsh in all of Wales. As defined in equation (2.5), *p_B_* in figure 2 corresponds to the population fraction able to speak the minority language (either as monolinguals or bilinguals). In particular, for the languages spoken in the UK, figure 2*a,c,d*, *p_B_* corresponds mostly to bilinguals, because monolingual speakers are very low in number and become extinct during the considered period (see, e.g., the supplementary material in reference [30]). In the case of the data on the Quechua language, the number of bilinguals is not recorded officially [32]; however, it is considered to be low, and bilinguals tend to insist in their children becoming Spanish monolinguals [41], thus preventing an effective increase of bilingual individuals.

The parameter values that yield the best fit for each language dataset are indicated in figure 2. The value of the sum of squared errors, *χ*^2^, is presented in each plot as an estimation of the fitting error. Figure 2 shows that the new model provides a good fit to the experimental data, as shown by the low values of *χ*^2^. Our model (equation (2.5)) has three adjustable parameters (*γ*, *α* and *β*). Similarly, the model by Abrams and Strogatz (equation (1.1)) also has three adjustable parameters (*γ*, *α* and *s*). However, the sums of squared errors *χ*^2^ for our model (figure 2) never exceed those obtained from fitting the Abrams–Strogatz model to the data (the best fits of the data to the Abrams–Strogatz model yield 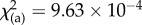, 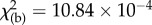, 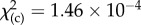, 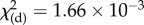). Therefore, the model given by equation (2.5) agrees with the observed data at least as well as the Abrams–Strogatz model (equation (1.1)). Our model, however, does not present the limitations detailed in §2.1 when predicting the language shift with no protection policies—namely the need in some cases of a significantly large fraction of high-status speakers for the shift to happen, or the prediction of a rather improbable equilibrium situation when a minority language is nearly extinct.

Comparing the values of the parameters obtained in figure 2, we see that we obtain similar values for the three datasets for Celtic languages in the UK (figure 2*a*,*c*,*d*), whereas the parameter values for the Quechua language differ significantly from them (figure 2*b*). This is a rather reasonable result, because Scottish Gaelic and Welsh have evolved in rather similar conditions, which may easily differ from the situation of the Quechua language. There is, however, a remarkable difference in the value of *α* in the region of Monmouthshire and when considering the whole of Wales, which shows a higher resistance to change in the area of Monmouthshire. This may be due to the fact that, at least nowadays, Monmouthshire is a rather rural area (as most of Wales). By contrast, the data for all of Wales also include the most densely populated areas and big cities (with a 50% of the total population residing in a 10% of the total area of Wales [42]), where the language shift tends to take place at a faster rate [14].

### 3.2. Language replacement fronts

**Figure 3.**
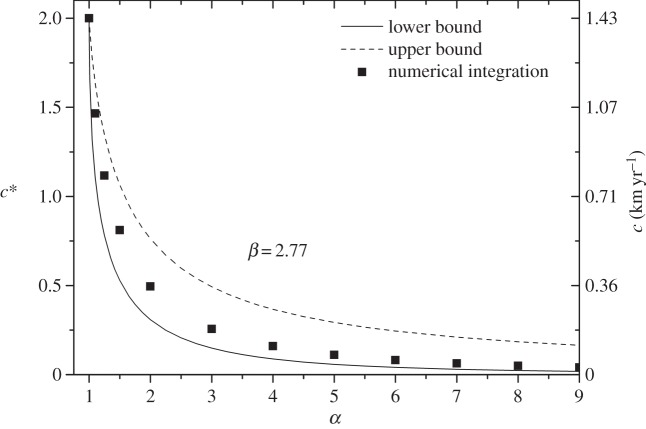
Sensitivity of the dimensionless speed with the parameter *α*. Lines correspond to the upper (dashed) and lower (solid) bounds for the front speed. Symbols correspond to the numerical simulation. All results are calculated for the mean value *β* = 2.77. The dimensional speed (right axis) has been calculated assuming *γ* = 0.1 yr^−1^ and *D* = 5.08 km^2^ yr^−1^.

**Figure 4.**
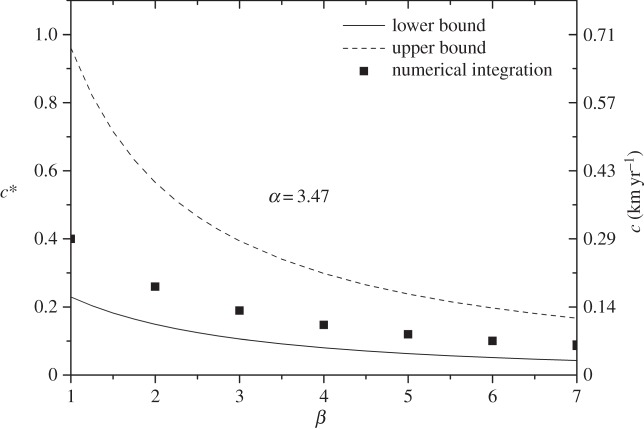
Sensitivity of the dimensionless speed with the parameter *β*. Lines correspond to the upper (dashed) and lower (solid) bounds for the front speed. Symbols correspond to the numerical simulation. All results are calculated for the mean value *α* = 3.47. The dimensional speed (right axis) has been calculated assuming *γ* = 0.1 yr^−1^ and *D* = 5.08 km^2^ yr^−1^.

Figures 3 and 4 show the results of computing the speed of the linguistic front for several values of *α* (figure 3) and *β* (figure 4). In both plots, the lines correspond to the lower (solid) and upper (dashed) bounds for the front speed, calculated with equations (2.11) and (2.14), respectively. The symbols are the results of the numerical integration of system (2.7) and they fall, as expected, within the analytic range. All of the speeds have been computed using dimensionless units (left axis), and the right axis corresponds to the dimensional speed (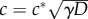) for an example with *γ* = 0.1 yr^−1^ (figure 2*a*) and *D* = 5.08 km^2^ yr^−1^. This value of the diffusion coefficient has been estimated from 
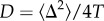
 [43] and observed values from modern populations for the generation time (*T* = 25 yr [25]) and the mean-squared displacement (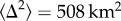 [44,45]). The latter was obtained from modern human populations in the Parma Valley, Italy, during the twentieth century, and is therefore coetaneous with the data in the studied period (figure 2).

In figures 3 and 4, we study the sensitivity of the model to the parameters *α* and *β*. In figure 3, the results have been computed using a fixed value of *β* = 2.77, the mean of the values obtained from the fits in figure 2, and for the range of *α* also obtained from the best fits in figure 2, 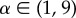. Similarly, in figure 4, the results have been plotted for the mean value *α* = 3.47 and the range *β ∈* (1, 7), also obtained from figure 2. For both parameters, *α* and *β*, the dimensionless speed decreases (and so does the dimensional speed if considering a fixed value of *γ*) for increasing values of these parameters (because this corresponds to less language conversions per unit time, see equation (2.5) and recall that *p_i_* ≤ 1 for *i* = *A*,*B*). However, we see that the front speed is more sensitive to variations of *α* than to those of *β*. In addition, for *α* = 1, we find that the lower and upper bounds and the numerical integration always converge at c* = 2.

Because the value of the dimensional speed depends on the value of *γ*, in table 1, we show the dimensionless and dimensional speeds computed for the four datasets in figure 2, as well as the parameter values obtained from figure 2 and used to compute these results (we use the same labelling for the languages as in figure 2). The dimensional speeds in table 1 have been calculated using two possible values of the diffusion coefficient: *D* = 5.08 km^2^ yr^−1^ (as in figures 3 and 4), obtained from mobility data on Italian populations in the twentieth century [44,45] (see above), and *D* = 6.72 km^2^ yr^−1^, obtained from mobility data in the eighteenth–nineteenth centuries in Catalonia, Spain [46]. The first estimate is probably a better approach for the twentieth-century European languages, because they are coetaneous. We do not have estimates for South American populations, but because the Quechua-speaking communities are very traditional agricultural communities [16], the second estimate, corresponding to a more traditional agricultural society, might be a better approach. However, both values give similar results for the front speed (table 1).

**Table 1.** Parameter values and predicted dimensionless and dimensional speeds of linguistic fronts. Dimensional speed *c*_(1)_ corresponds to a *D* = 5.08 km^2^ yr^−1^, and *c*_(2)_ to *D* = 6.72 km^2^ yr^−1^. The language labels correspond to the same labels used in figure 2.

minority language	*α*	*β*	*γ* (yr^−1^)	c*	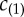 (km yr^−1^)	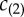 (km yr^−1^)
(a) Scottish Gaelic, Sutherland	1.73	1.56	0.099	0.742	0.526	0.605
(b) Quechua, Huanuco	8.93	6.26	426.32	0.0065	0.302	0.348
(c) Welsh, Monmouthshire	2.23	1.76	0.237	0.508	0.557	0.641
(d) Welsh, all of Wales	1.00	1.48	0.029	2.00	0.768	0.883

For the case of Welsh, the observed front speed was estimated in reference [25] from language distribution maps to be within the range 0.3 − 0.6 km yr^−1^. Comparing this observed range with the model predictions for the Welsh retreat front (table 1(c) and (d)), we see that the observed speed range is consistent when using the data for (c) Welsh in Monmouthshire, whereas the estimates are somewhat faster when using the data for (d) Welsh in all of Wales. We stress again that, at least at present, half the population of Wales is concentrated in 10% of its area (in the southern coast, near Cardiff), whereas the rest is rural and approximately equally populated [42]. Thus, whereas it is very possible that the language shift dynamics are different in large agglomerations than in rural areas, to obtain a realistic estimate for the speed of the linguistic retreat in the whole of Wales, it might be more reasonable to use the data from Monmouthshire, because it may be representative of a larger (rural) area. In such a case, we can, indeed, consider that there is a good consistence between model and observations.

We do not have estimates for the observed speeds of retreat for (a) Scottish or (b) Quechua, but their predicted dimensional ranges have the same order of magnitude and similar ranges to those reported for the Welsh language (table 1).

## 4. Discussion

In this paper, we have introduced a new model to explain historical data on the decline of minority languages when in competition with other languages which are perceived as more advantageous. We have fitted our model (equation (2.5)) to historical data, and it yields reasonably good fits (figure 2), as good as or better than the Abrams–Strogatz model (equation (1.1)). A significant feature that can be observed from these fits is the fact that the three datasets corresponding to endangered languages in the UK present similar parameters, probably owing to having endured similar conditions, whereas they differ significantly from the estimates corresponding to the evolution of the Quechua language in Peru.

In our model, we have considered only two populations, speakers and non-speakers of the endangered language *B*, without explicitly considering bilinguals (they are included as *B*-speakers), in contrast to models prepared by some other authors [21,30,31]. This is partly due to practical reasons, because we are applying our model to the same datasets as in reference [19]. But besides this fact, we consider that dividing the population in speakers and non-speakers of a minority language is a good enough division to establish the language health and estimate the evolution of this minority language if its perceived value does not change.

In addition, our model does only allow for the high-status language to gain speakers and eventually become the only available language, whereas other authors have found bilingualism as a possible stable outcome [30,31]. However, in these models, the social status of the endangered language was allowed to change owing to language policies [30], or the data used corresponded to a period where those policies had already started to efficiently change the status of the minority language [31]. Then, the fact that bilingualism may be a viable solution does not disagree with our model. Indeed, according to linguistic studies, stable bilingualism is only possible when the status of the minority languages is raised [14,47], whereas our model shows the predictions if no such change occurs.

It is worth, therefore, noting that while in the case of Scottish Gaelic no language policies were applied during the considered period [28], this is not the case for the other two languages. Quechua was made official in Peru in 1975, although none of the bilingual programmes created to maintain it have been able to effectively improve its status and stop the language shift [48]. By contrast, the linguistic policies applied in Wales since the 1970s seem to have been able to raise the status of Welsh and stabilize (see figure 2*d* after 1971) and even reverse the languages shift (after 2001 [30,42]). In that sense, the data for the Welsh language after 1971 should probably have been omitted from the analysis, but because there was still language loss (figure 2*d*), fits do not show significant divergence in the parameter estimates with or without these data points.

So, in an attempt to model language competition when one of the languages is at a clear disadvantage against another language seen as more advantageous, in our model, equation (2.5), language shift is allowed in only one direction (i.e. from the less attractive to the more attractive language). Other authors did allow a reverse flow of speakers between languages, with their relative importance determined by a status parameter [19,30]. However, even though a double-sense flow may seem reasonable for competing languages with similar conditions, a unidirectional flow agrees with the behaviour observed in many societies, in which local speakers cease to transmit their language to their children [15,17]. Moreover, recent historical data suggest that, at least when the high-status language is widely regarded as advantageous, such as English or Spanish (the high-status languages corresponding to the datasets in figure 2), and no language-planning policies are applied: (i) speakers of the high-status language (almost) never learn the indigenous language [12,27]; (ii) once the replacement process is started, it tends to gradually run its course towards total substitution (unless political measures are taken) [15–17]; and (iii) even when the initial number of speakers of the minority languages is high, it can become extinct in a time interval as short as one generation [17]. In fact, the Abrams–Strogatz model (equation (1.1)) presents two important limitations: (i) if *α* > 1 it predicts that, for large enough values of the initial proportion of speakers of the low-status language, then high-status speakers shift their language and use the low-status one instead (which would stop the observed language decline); (ii) if *α* < 1 it predicts that, when the proportion of speakers of the high-status language becomes large enough, again high-status speakers shift their language and use the low-status one instead (which seems strange and, again, has not been observed). By contrast, the language shift model that we have used in this paper does not have these limitations. In addition, the results in figure 2 show that our model (equation (2.5)) can, indeed, give a good account of the observed trends in the decline of minority languages.

In this paper, we have also explored the geographical aspects of language shift, particularly the rate at which a more advantageous language expands geographically and replaces the indigenous one. Besides the mathematical study of the sensitivity of the model to the parameters, we have also calculated and assessed the front speed obtained from the datasets in figure 2. We have seen that the predicted speed for the Welsh retreat is consistent with the observational estimation (0.3 − 0.6 km yr^−1^ [25]). We do not have observed estimates for the other two languages, but because they lie within the same order of magnitude, they may also be realistic predictions.

In addition, besides the numerical integration approach, we have been able to obtain mathematical expressions for lower and upper bounds to the rate of spread, equations (2.11) and (2.14). The usefulness of these bounds is, on the one hand, that they provide a check of the numerical results; on the other hand, they make it possible to perform quick estimates, without discretizing the set of differential equations and performing numerical integrations on a grid.

To obtain the estimates on the front speed, we have applied the parameters obtained from the non-spatial model to the spatial approximation. This is a realistic assumption, because when integrating the spatial model, equation (2.8), over the whole region, we should obtain the total variation corresponding to the non-spatial model, equation (2.5). This might be affected if there was a high migration towards or from the studied region. However, according to statistical data in the UK [35], the migration rates have increased in the past 20 years, and even in such conditions, the immigration rate is about an order of magnitude lower than the language shift rate in figure 2. For the Peruvian case, because the indigenous communities are generally poor- and tight-knit communities, high migration rates are rather unlikely [16]. In future work, however, it could be interesting to fit time curves at different places to estimate the parameters *α*, *β* and *γ* as a function of the position, and then use them to predict the front speed also as a function of the position.

In addition, note that the case in which high-status speakers move mainly to urban areas (that then act as hub) does not correspond to a front propagating across a homogeneous geography. Therefore, in such cases, it would be necessary to develop a different model which could be the subject of future work.

Finally, we would like to note that the models developed in this paper could be applied also to other processes of cultural transmission where the cultural trait being transmitted can be described as a clear advantageous change (at least from the subjective perspective of the prospective receivers).

## Endnote

1 Note that according to reference [19], the population groups are monolingual, but comparing their data with census data (e.g., see supplementary information in reference [30]) one can induce that, at least for the British languages, the bilingual population is included in *p*_B_.
